# Optimum aquaculture and drying conditions for W*olffia arrhiza* (L.) Wimn

**DOI:** 10.1016/j.heliyon.2023.e19730

**Published:** 2023-09-03

**Authors:** Kakanang Prosridee, Ratchadaporn Oonsivilai, Arak Tira-aumphon, Jittra Singthong, Jirawan Oonmetta-aree, Anant Oonsivilai

**Affiliations:** a School of Food Technology, Institute of Agricultural Technology, Suranaree University of Technology, Nakhon Ratchasima, 30000 Thailand; b Health and Wellness Research Group, Suranaree University of Technology, Nakhon Ratchasima, 30000 Thailand; c School of Plant Production, Institute of Agricultural Technology, Suranaree University of Technology, Nakhon Ratchasima, 30000 Thailand; d Department of Agro-Industry, Faculty of Agriculture, Ubon Ratchathani University, Warinchamrap, Ubon Ratchathani, 34190 Thailand; e Food Science and Technology Program, Faculty of Science and Technology, Nakhon Ratchasima Rajabhat University, Muang, Nakhon Ratchasima, 30000 Thailand; f School of Electrical Engineering, Institute of Engineering, Suranaree University of Technology, Nakhon Ratchasima, 30000 Thailand

**Keywords:** Aquaculture, Drying, Duckweed, Flavonoids, Nutritional, Phenolics, Protein

## Abstract

This study aimed to investigate the effects of aquaculture and the optimum conditions for drying duckweed plants to maintain the highest nutritional value and bioactive substances. Protein quantification was used to screen duckweed plants subjected to the 14 treatments under aquaculture conditions. Proximate analysis of three aquaculture conditions showed the highest quantification of protein. Moreover, these samples were analyzed for total phenolics, flavonoids, and chlorophylls. The optimal drying conditions for duckweed plants with the highest protein content were determined using a factorial design with three temperature and time parameters.

The results showed that the duckweed under aquaculture conditions in an outdoor cement pond with hydroponic electrical conductivity (EC) of 0.5 mS/cm contained the highest protein at 41.81 ± 3.40%. Moreover, proximate analysis of this sample showed fat, fiber, moisture, ash, and carbohydrate contents of 1.99 ± 0.08%, 4.46 ± 0.71%, 3.29 ± 0.17%, 22.06 ± 0.07% and 14.12 ± 1.63%, respectively. In addition, the optimum drying conditions for this sample were 50 °C and a drying time of 6 h. Under optimum drying conditions, this sample showed total phenolics, flavonoids, and chlorophylls contents of 55.28 ± 1.35 (μg GAE/g dry weight), 159.84 ± 6.65 (μg catechin equivalent [QE]/g dry weight) and 22.91 ± 0.15 (mg/g dry weight), respectively.

In conclusion, the dried duckweed under aquaculture conditions in an outdoor cement pond with hydroponic EC 0.5 mS/cm contained the highest contents of proteins, total phenolics, total flavonoids, and total chlorophyll, which could be used as functional ingredients in health food products.

## 1 Introduction

Plant-based proteins have recently attracted considerable attention as alternatives to animal proteins. Because plant-based proteins have a lower environmental impact and can be produced more efficiently than animal proteins, they can be used to address future food problems and inadequate protein intake. Furthermore, they are expected to provide various health benefits.

Niyanuch and Tangwongchai (2010) studied the chemical, physical, and microbial composition of fresh duckweed, *Wolffia arrhiza* (L.) Wimm. Fresh duckweed was found to contain 24.31% protein, 3.04% fat, 12.68% coarse fiber, 11.05% insoluble fiber, 3.82% soluble fiber, 14.87% total dietary fiber, and 19.97% ash by dry weight. Moreover, it also showed 30.17 mg/100 g total chlorophyll, 3.43 mg/100 g beta-carotene, 0.40 mg/100 g riboflavin, and 21.14 mg/g total phenolic substance contents by dry weight. Finally, the fresh duckweed showed antioxidant activities of 65.91% using the ABTS method and 70.12% using the DPPH method [1].

Duckweed is a rare commodity. Most duckweed plants are found by accident, or as a result of knowing that they were harvested from the same water source where they were previously found. Duckweed cannot grow, or grows in diminished quantities, in the presence of contamination. Duckweed grows best in environments with good environmental control. This results in better growth of duckweed and a higher nutritional value [2].

Damna et al. [2] studied the effects of combinations of three fertilizer types (goat manure [2 kg/m^2^], chemical fertilizer [100 mg/L], and hydroponic fertilizer [EC 0.5 mS/cm]) and three light filter levels (no filter, 50% light intensity with a one-layer filter, and 50% light intensity with a two-layer filter) to determine their effect on the growth and yield of *Wolffia*. A 3 × 3 factorial experiment was conducted using a completely randomized design with three replicates. *Wolffia* were cultured in a cement tank (80 cm diameter) in which the water level was controlled at 20 cm, pH was adjusted to 5–6, plants were harvested 20 days after culture, and their growth and yield data were recorded and analyzed. The results showed highly significant differences (P < 0.01) in fresh weight, dry weight, color, chlorophyll content, and protein content of *Wolffia*. Hydroponic fertilization with no light filter resulted in the highest fresh weight, dry weight, and chlorophyll contents (1236.49 g/m^2^, 38.80 g/m^2^ and 41.64 mg/100 g, respectively). The highest brightness and yellow color were obtained with the chemical fertilizer at all light filter levels. Hydroponic fertilizer with 50% light intensity and a two-layer filter yielded the highest protein content (40.46%). These results could be used to improve the quality of *Wolffia* production.

Zhubin et al. [3] investigated the nutritional properties of a high-protein strain of duckweed, *Wolffia arrhiza* 7678a, which had a protein content of 50.89% of dry weight. The study found that this duckweed strain has good quality protein, with a digestible indispensable amino acid score of 0.75. Furthermore, it contains a high proportion of unsaturated fatty acids (FAs), which are considered healthier than saturated FAs and possess antioxidant properties. Compared with conventional crops, it has higher levels of total phenolics and flavonoids, making it a potentially valuable source of antioxidants. Finally, low antinutritional factor concentrations in *Wolffia arrhiza* were easily absorbed. These results suggest that nutrients can be easily absorbed by the body, indicating their potential as a human food source.

Ketkaew and Rakthai [4] examined the light intensity and light period for *Wolffia globosa* production. The methodology of this research selected the light intensity level (4,000, 8,000, 12,000, 16,000, and 20,000 lx) and light period (8, 10, 12, 14, and 16 h per day) that affected the maximum marginal yields. The results showed that the optimum light structure for the production of the aquatic macrophyte (*Wolffia globosa*) was a light intensity of 12,000 lx with 14 h of light per day, which produced the maximum marginal aquatic macrophyte yield (295.20 ± 17.96 g) and aquatic macrophyte dry weight yields (11.44 ± 0.66 g).

Piotroska et al. [5] studied the influence of exogenously applied jasmonic acid (JA) at different JA concentrations on the growth and metabolism of *Wolffia arrhiza* (*Lemnaceae*). The results revealed that the effects of JA were concentration-dependent, with 0.1 M JA promoting plant growth and improving *Wolffia arrhiza* viability, while 100 M JA had detrimental effects on plant metabolism and growth. This study also highlights the role of JA in regulating enzymatic and non-enzymatic antioxidant systems in *Wolffia arrhiza*, thus suppressing lipid peroxidation. These results suggest that JA can improve the growth and viability of aquatic plants, including *Wolffia arrhiza*, and can be used to enhance their production.

Suppadit et al. evaluated the use of *Wolffia arrhiza* meal (WAM) as a protein replacement for soybean meal in the diet of laying Japanese quails. The results showed that the inclusion of WAM in the diet had no negative effects on the performance or egg quality characteristics of the quails. Moreover, egg protein content increased because of WAM and the feed cost per kilogram of eggs produced decreased [6].

Previous research has analyzed the FA profiles of 30 duckweed species to assess the natural diversity within the *Lemnoideae* family. The study found that the total FA content varied among different species, ranging from 4.6% to 14.2% of dry weight, whereas triacylglyceride varied from 0.02% to 0.15% of dry weight. Additionally, this study identified three FA (palmitic, linoleic, and-linolenic acids) that comprised more than 80% of the total duckweed FA. Seven *Lemna* and two *Wolffiella* species accumulated polyunsaturated FA containing Δ6-double bonds, including gamma-linolenic acid (GLA) and stearidonic acid (SDA). This study also identified a putative Δ6-desaturase gene, designated LgDes, in the DNA sequence of *Lemna gibba*. Expression of a synthetic LgDes gene in *Nicotiana benthamiana* resulted in the accumulation of GLA and SDA, confirming that it specifies a Δ6-desaturase. Overall, this study provides insights into the potential of *Lemnoideae* as a source of biofuel or as a dietary supplement for humans and animals because of the natural diversity of their FA profiles [7].

A previous study aimed to develop a suitable culture system for the mass production of *Wolffia globosa* for human consumption. The results showed that the most suitable system was horizontal surface agitation, as it provided the highest mass of 42.94 ± 2.17 g/m^2^ and a yield of 1.52 ± 0.04 g dry weight/m^2^/d, which was significantly higher than the yields of the other systems. Additionally, the biomass of *Wolffia globosa* contained 48.2% protein with complete essential amino acids, 9.6% fat, and 14.5% crude fiber, with low bacterial contamination, making it a potential protein source for human consumption. Overall, this study provides useful insights into the cultivation of *Wolffia globosa* for mass production and its potential as a food source. These results suggest that horizontal surface agitation is a suitable method for the mass production of *Wolffia globosa*, which could be used as an alternative protein source for human consumption [8].

In 2022, Kumla et al. [9] investigated the potential of Wolffia sp. to treat wastewater generated from Climbing Perch cultures. The results showed that the use of Wolffia sp. biomass could effectively reduce biological oxygen demand, ammonia, total suspended solids, total nitrogen, and total phosphorus levels in wastewater, while also regulating the pH and dissolved oxygen levels suitable for freshwater fish culture. However, this study also revealed that an excessive amount of biomass can result in death, leading to the release of organic nitrogen, which can be reused as inorganic nitrogen for further growth. Therefore, the use of Wolffia sp. in wastewater treatment could produce valuable nutrients that can be used in other industries.

The study by Appenroth et al. investigated the nutritional value of 11 species of the genus *Wolffia* traditionally used as human food in Asian countries. The results showed that *Wolffia* species contained high levels of essential amino acids, polyunsaturated FAs, and minerals. Additionally, *Wolffia microscopica* showed the highest yield for most nutrients and had a rapid growth rate, indicating its potential for practical applications in human nutrition. This study provides valuable insights into the nutritional value of *Wolffia* species and their potential use as food sources. Further research is required to optimize cultivation methods and assess the safety and nutritional value of *Wolffia* species for human consumption [10].

At present, the optimum conditions for the growth and drying of *Wolffia* have not been studied, particularly the nutritional value and bioactive substances when duckweed is incubated in different production methods, such as various containers and light intensities, compared with commercial products. These results would provide scientific information on the optimum aquaculture conditions to obtain high nutritional value and bioactive substances such as total phenolics, chlorophyll, and flavonoids. Therefore, to increase the nutritional value of duckweed, this study investigated the optimum aquaculture conditions and drying parameters for duckweed, based on its nutritional value and bioactive substances.

## 2 Materials and methods

### 2.1 Sample preparation

For duckweed production, for samples 1–5 and 7–9, a vertical tray with a dimension of 0.9 × 1.0 × 0.4 m was applied. In addition, for sample 6, a cylindrical horizontal stirring system with a propeller and a volume of 0.01 m^3^ was used. for samples 10–13, a cement pond with dimension of 1.0 × 0.4 × 0.4 m was used. The commercial product was duckweed bought from a supermarket in Nakhon Ratchasima Province from March to April 2021[Table 1].

**Table 1:** Details of how 14 fresh duckweed plants were cultured to one-week maturity.

No.	Detail
No.1	Indoor – vertical tray type, light intensity 200 μmol/m^2^/s fluorescent white light
No.2	Indoor – vertical tray type, light intensity 250 μmol/m^2^/s LED white light
No.3	Indoor – vertical tray type, light intensity 200 μmol/m^2^/s LED white light
No.4	Indoor – vertical tray type, red + blue LED light intensity 200 μM/m^2^/s
No.5	Indoor – vertical tray type, white + red + blue LED light intensity 200 μM/m^2^/s
No.6	Indoor – cylindrical horizontal stirring system, LED light, concentrated light 200–600 μM/m^2^/s
No.7	Indoor – vertical tray type, water supply
No.8	Outdoor – vertical tray type hydroponics fertilizer EC 0.5 mS/cm
No.9	Outdoor – vertical tray type, reverse osmosis water
No.10	Outdoor – cement pond – manure – pig manure
No.11	Outdoor – cement pond – chemical fertilizer formula 16-16-16
No.12	Outdoor – cement pond – hydroponics fertilizer EC 0.5 mS/cm
No.13	Outdoor – cement pond – hydroponics fertilizer EC 1 mS/cm
No.14	Commercial sample (control)

Experimental design.

This study used the highest protein content as the main parameter for selecting the aquaculture conditions for further investigation of drying conditions using a hot-air oven [Table 2]. Other parameters such as total phenolics, flavonoids, and chlorophyll were also considered to identify the optimum aquaculture conditions. Fresh duckweed was dried in a hot-air oven at 60 °C for 6 h [3] and then crushed using a size-reduction machine. Then, it was sieved through a 100 mesh sieve (149 μm), packed into plastic bags, and stored in a desiccator.

**Table 2:** Conditions for drying duckweed samples in each of the nine experiments.

Sample	Temperature (°C)	Time (h)
**1**	50	4
**2**	60	4
**3**	70	4
**4**	50	5
**5**	60	5
**6**	70	5
**7**	50	6
**8**	60	6
**9**	70	6

An experiment was designed to determine the optimum conditions for drying duckweed samples using a factorial experimental method.

### 2.2 Proximate composition analysis

The chemical composition of dried duckweed was studied by analyzing the moisture (AOAC Method 925.10), ash (AOAC Method 900.02 A), protein (AOAC Method 928.08), fat (AOAC Method 945.16), crude fiber (AOAC Method 978.10), and carbohydrate (AOAC Method 995.13) contents [11].

### 2.3 Bioactivity

#### 2.3.1 Total phenolic content

Total phenolic content was determined using the Folin–Ciocalteu procedure previously described by Oonsivilai et al. [12,13] and Thaiudom et al. [14], and gallic acid (Sigma-Aldrich Co., MO, USA) was used as a standard. Aliquots (0.02 mL) of the gallic standard/sample/blank were transferred to test tubes. After 0.1 mL Folin–Ciocalteu reagent was added, and the solution was mixed and allowed to stand for 5 min. Next, 0.3 mL of 20% (w/v) Na_2_CO_3_ was added, and tubes were vortexed and stored in the absence of light for 120 min at room temperature for comparison with a gallic acid standard. The absorbance was measured at 765 nm using a spectrophotometer (Libra S22 S/N 97765, Biochrom, Cambridge, UK). The results are expressed as gallic acid equivalents.

#### 2.3.2 Total chlorophyll content

Dried duckweed (0.05 g) was weighed after using a 100 mesh sieve, and then placed in a 50 mL centrifuge tube along with 10 mL of an 85% by volume (% v/v) acetone solution and 0.1 g magnesium carbonate. Then, the mixture was centrifuged at 10,000×*g* for 10 min at 4 °C, while the final volume was adjusted to 25 mL with acetone solution and then analyzed for total chlorophyll content. The absorbance was measured using a spectrophotometer at wavelengths of 645 and 663 nm. The analysis results are reported in mg/g of the sample, and the total chlorophyll content was calculated according to the following formulae [12].

Chlorophyll *a* = [12.7(A663) – 2.69(A645)] x V/(1000xW)

chlorophyll *b* = [22.9(A645)-4.68(A663)] x V/ (1000xW)

Total chlorophyll = chlorophyll *a* + chlorophyll *b*.

Note: A = Absorbance (Abs)

V= Volume of extract (mL)

W= Weight of sample (g)

#### 2.3.3 Total phenolic content

The total phenolic contents were investigated using the aluminum chloride colorimetry procedure [15] and by dissolving the dry extract with solvent, water, or ethanol, pipetting 250 μL of each sample solution, then placing it in a test tube and adding 1.25 mL of deionized (DI) water and 75 μL of 5% NaNO_2_. The solution was then mixed thoroughly and set aside for 15 min. Then, 10% AICI_3_ 150 μL, 1 m NaOH 0.5 mL, and 275 μL DI water were added, mixed thoroughly, and set aside for 5 min. Absorbance was measured at 510 nm to determine total flavonoid content using catechin as the standard. The standard concentrations used were 1.56, 3.12, 6.25, 12.5, 25, and 50 μg/mL in water. The results of the analyses were reported in mg of catechin equivalents (CE)/g of the sample.

### 2.4 Chromatography conditions for polyphenol analysis

Polyphenol *chromatography was performed as described by Oonsivilai* et al. [13] *using* reverse phase (4.6 mmI.d. × 250 mm) and a C18 column with a guard column *containing the same stationary phase (Grace Vydac, CA, USA)*. *The flow rate was 1.*0 mL/min at 35 °C*.* The binary mobile phase comprised a water:acetic ratio of 98:2 v/v (reservoir A) and acetonitrile (reservoir B). An initial ratio of 99:1 v/v (A/B) to 70:30 v/v was used for 20 min. *The gradient was held for* 10 min*, followed by a* 5 min *linear gradient back to 100% (A) and equilibration at the initial condition for* 5 min *for a total run time of* 30 min*.* Phenolics were detected using a photodiode array detector with an absorbance between 200 and 500 nm. The reference phenolic acid standards comprised caffeic, *p*-coumaric, ferulic, gallic, protocatechuic, rosmarinic, and sinapic acids. Reference standard stock solutions of gallic, protocatechuic, caffeic, and *p*-coumaric acids were prepared by accurately weighing and dissolving the acids in DI water. The ferulic and sinapic acids were dissolved in 95% ethanol to obtain the final concentration of 500 μg/mL. Then, the stock solutions were diluted to a range of 1–500 μg/mL. Calibration curves were established to quantify phenolic acids.

## 3 Results and discussion

### 3.1 Chemical composition

Table 3 lists the obtained experimental results. When the duckweed samples were cultured under different conditions, the results showed that a % yield was obtained in the range of 3.511–8.674%. Previous research [4] reported a % yield of dried duckweed of 4.19–4.80%, which was produced with a light intensity of 4000–20,000 lx for 14 h per day. Protein content was determined using Kjeldahl analysis to determine the top 3 oocyte samples with the highest protein content from the 14 different cultured samples. Samples 10,0.6, and 12 had the highest protein contents of 61.702, 54.884, and 54.079%, respectively. The content varied according to the different conditions. These results were higher than those of a study by Zhubin et al. [3] that showed a 50.89% protein content of duckweed, but lower than that of Damna et al. [2] of 40.46% produced using hydroponic fertilizer (EC 0.5 mS/cm, NO3^-^ 237.84, NH4^+^ 30.75, H2PO4^-^ 30.04, K 290.00, Ca 113.56, Mg 30.00, SO_4_^2−^ 40.00, Fe 2.4, Mn 1.63, Cu 0.125, Zn 0.44, B 0.51, Mo 0.0225 mg/L) with a two-layer light filter.

**Table 3:** The % yield of all 14 dried duckweed samples, dried at 60 °C for 6 h with a hot air dryer (tray drier), after 1 week of incubation.

No.	% Yield	% Protein
No.1	5.334 ± 0.01^f^	50.715 ± 0.14^ef^
No.2	5.573 ± 0.01^h^	49.277 ± 0.43^e^
No.3	5.364 ± 0.01^f^	51.725 ± 0.47^f^
No.4	5.495 ± 0.01^g^	49.310 ± 0.30^e^
No.5	3.511 ± 0.01^a^	49.124 ± 0.73^e^
No.6	4.592 ± 0.01^b^	54.884 ± 0.67^g^
No.7	8.674 ± 0.01^k^	15.928 ± 0.90^a^
No.8	5.130 ± 0.01^e^	43.915 ± 0.61^c^
No.9	5.689 ± 0.05^i^	25.520 ± 0.51^b^
No.10	4.594 ± 0.06^b^	61.702 ± 0.97^h^
No.11	4.888 ± 0.05^d^	45.540 ± 0.48^d^
No.12	4.703 ± 0.06^c^	54.079 ± 0.57^fg^
No.13	5.333 ± 0.03^f^	49.102 ± 0.34^e^
No.14	8.170 ± 0.02^j^	25.480 ± 1.05^b^

*Results are shown as the mean ± standard deviation of three iterations (n = 3) with different letters a–h in the same column (P < 0.05) (Duncan's New Multiple Range Test).

From Table 4, we can compare the protein content obtained from the three samples with the highest protein content from the experiment in Table 3, which shows the protein content obtained using the Kjeldahl and the Biuret methods, with sample No.14 as a control because it is a natural duckweed. The results showed that the protein content analyzed using the Kjeldahl method was higher than that analyzed using the Biuret method. This is because Kjeldahl analysis uses a technique to analyze the total nitrogen content of a sample. Consequently, this also includes the manure used for farming. Therefore, sample No.10, which was cultured with manure and pig manure, had the highest protein content, as determined by Kjeldahl analysis. Biuret analysis tests for substances that consist of two or more peptide bonds. Compounds containing at least three amino acids and proteins yielded a purple solution. This directly indicates the protein content of the sample; Therefore, samples analyzed using the Biuret method must be selected. This is the simplest and most rapid method for detecting proteins in a sample. It provides a stable color; hence, it does not cause deviations like other methods such as UV absorption and the Lowry method. Moreover, apart from proteins, very few compounds interfered with the test. It only identifies nitrogen from proteins or peptide bonds. This indicated that non-protein nitrogen was not detected. The samples with the highest protein contents analyzed using the Biuret method were No.12, No.10, and No.6, with protein contents of 41.806, 35.454, and 25.557%, respectively. The protein contents of samples 6 and 14 were not significantly different. The sample with the highest protein content was selected for further analysis. Sample 12 was used to test the drying conditions at different temperatures and times.

**Table 4:** The % of protein per dry sample weight of the duckweed dried at 60 °C for 6 h, with No.14 as the control.

Sample	% Protein^KM^	% Protein^BM^
No.6	54.884 ± 0.67^b^	25.557 ± 1.03^a^
No.10	61.702 ± 0.97^c^	35.454 ± 2.22^b^
No.12	54.079 ± 0.57^b^	41.806 ± 3.40^c^
No.14	25.480 ± 1.05^a^	20.550 ± 5.29^a^

Note: *Results are shown as the mean ± standard deviation of three iterations (n = 3) with different letters a–h in the same column (P < 0.05) (Duncan's New Multiple Range Test).

KM denotes the % of protein analyzed by the Kjeldahl Method.

BM denotes the % of protein analyzed by the Biuret Method.

Table 5 shows the preliminary chemical compositions obtained from a comparison of the three samples with regard to their fat, crude fiber, ash, moisture, and carbohydrate contents with sample 14 as the control. The fat contents of the three duckweed samples were between 1.183 ± 0.14 and 1.997 ± 0.08%, which was lower than that of [3] at 6.07 ± 0.30%. Samples 10 and 12 had significantly different lipid contents (P < 0.05). There was no statistically significant difference (P > 0.05) in fat content between samples 6 and 10, or between samples 6 and 12. (P < 0.05). The crude fiber contents of the three samples were between 4.073 ± 0.73 and 4.835 ± 0.46%, which was not statistically significant (P > 0.05). The ash contents of all three samples were between 18.872 ± 0.07 and 25.351 ± 0.85%, with No.6 having the highest ash content, followed by No.12 and No.10. The moisture contents of the three samples were between 3.292 ± 0.17 and 4.750 ± 0.16%. There were no statistically significant differences (P > 0.05) in the moisture content of any of the three samples. In all three samples, the amount of carbohydrates was between 8.773 ± 1.50 and 14.119 ± 1.63%. Samples 6 and 10 had no statistically significant differences (P > 0.05) in carbohydrate content. Samples 12, 6, and 10 had statistically significant differences in carbohydrate content (P < 0.05). After drying, the samples were found to contain fat. The crude fiber, ash, and carbohydrate contents were high; however, the moisture content was low compared with the remaining two samples. The carbohydrate contents of the samples (8.77 ± 1.50–14.12 ± 1.63%) were lower than those reported in previous studies [1,3], which found carbohydrate contents ranging from 31.33 ± 0.27–33.85%. However, sample 14 (a commercial product) showed the highest carbohydrate content at 42.14 ± 1.68%. For the ash contents of the dried samples, samples 6, 10, 12, and 14 showed values ranging from 18.87 ± 0.07 to 25.35 ± 0.85%, which was higher than that reported by Ref. [3] at 11.71 ± 0.46%, but was similar to the value analyzed by Ref. [1] at 19.97%.

**Table 5:** The % of chemical composition (proximate analysis) per dry sample weight of three duckweed samples dried at 60 °C for 6 h, with No.14 as the control.

Sample	% Fat	% Crude fiber	% Ash	% Moisture	% Carbohydrate
**No.6**	1.560 ± 0.50^ab^	4.073 ± 0.73^b^	25.351 ± 0.85^d^	4.750 ± 0.16^b^	9.381 ± 0.37^a^
**No.10**	1.183 ± 0.14^a^	4.835 ± 0.46^b^	18.872 ± 0.07^a^	4.635 ± 0.16^b^	8.773 ± 1.50^a^
**No.12**	1.997 ± 0.08^b^	4.456 ± 0.71^b^	22.057 ± 0.70^b^	3.292 ± 0.17^a^	14.119 ± 1.63^b^
**No.14**	1.019 ± 0.22^a^	2.446 ± 0.15^a^	24.064 ± 0.71^c^	4.851 ± 0.25^b^	42.141 ± 1.68^c^

Note: *Results are shown as the mean ± standard deviation of three iterations (n = 3) with different letters a–h in the same column (P < 0.05) (Duncan's New Multiple Range Test).

The results showed that sample 12 had considerable nutritional value. With regard to the longest possible shelf life, sample 12 was the best of all 14 samples obtained from different cultures.

**Fig. 1:**
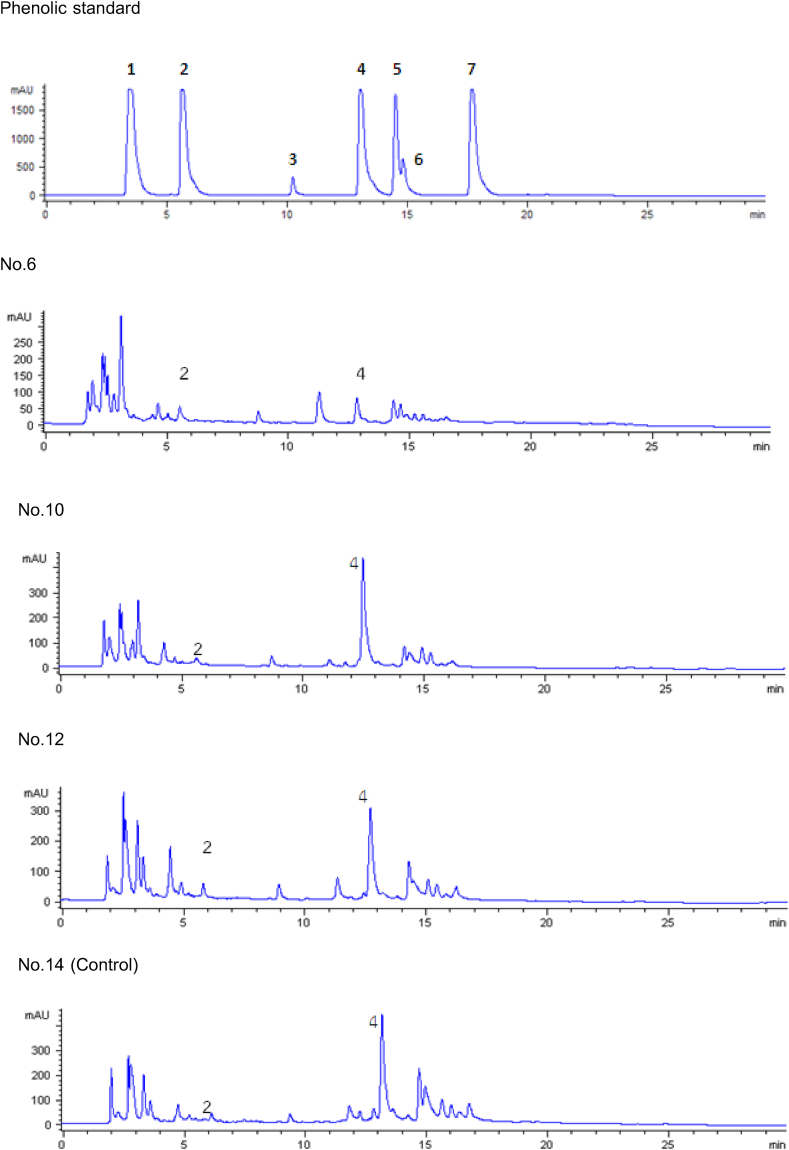
Chromatogram separation of phenolic compounds from standard phenolics and duckweed at a wavelength of 280 nm (1 = gallic acid, 2 = protocatechuic acid, 3 = caffeic acid, 4 = coumaric acid, 5 = ferulic acid, 6 = sinapic acid, and 7 = rosmarinic acid).

Fig. 1 shows the phytochemical compounds present in the duckweed. Duckweed samples 6, 10, 12, and 14 contained both the phenolic compounds. One type of phenolic compound included gallic, protochuic, caffeic, coumaric, ferulic, sinapic, and rosmarinic acids. The type and amount of phenolic compounds, including protochuic and coumaric acids, were the highest in duckweed. The protochuic acid contents of duckweed samples 6, 10, 12, and 14 were 1.94, 0.41, 1.38, and 1.20 mg/g raw material, respectively. The coumaric acid contents of duckweed samples 6, 10, 12, and 14 were 11.95, 76.72, 54.77, and 78.59 mg/g raw material, respectively. When duckweed is used as an ingredient in a prototype food product, products such as samples 10 or 12 should be used because they have the highest essence stability owing to a process that destroys minimal essence.

#### 3.1.1 Phenolic standard

Table 6 shows the moisture and free water contents of sample 12 when dried under different temperature and time conditions. The moisture contents of all nine conditions were between 1.794 ± 0.2 and 4.156 ± 0.26% of the samples obtained by drying under the same conditions. The moisture content was not significantly different among the groups (P > 0.05). However, samples obtained after drying for 4 and 5 h showed significant differences in moisture content when compared to the samples obtained after drying for 6 h (P < 0.05). Samples obtained by drying at the same temperature showed a statistically significant difference in moisture content (P < 0.05). The amount of moisture obtained depends on the drying time. The experimental results show that the samples obtained by drying under the conditions of 50, 60, and 70 °C for 6 h will achieve the lowest moisture contents between 1.687 ± 0.17 and 2.174 ± 0.42%. Moreover, the samples did not show any statistically significant differences (P > 0.05).

**Table 6:** The % of moisture and free water contents per dry sample weight of duckweed sample 12 which was dried under different temperature and time conditions in a tray drier.

Drying condition		% Moisture	Water Activity
Temperature (°C)	Time (h)		
50	4	4.008 ± 0.63^b^	0.411 ± 0.01^bc^
	5	3.502 ± 0.11^b^	0.408 ± 0.03^b^
	6	2.174 ± 0.42^a^	0.393 ± 0.03^ab^
60	4	4.156 ± 0.26^b^	0.464 ± 0.01^d^
	5	3.834 ± 0.23^b^	0.448 ± 0.01^d^
	6	1.687 ± 0.17^a^	0.374 ± 0.00^a^
70	4	3.942 ± 0.13^b^	0.448 ± 0.01^d^
	5	3.752 ± 0.87^b^	0.439 ± 0.01^cd^
	6	1.794 ± 0.27^a^	0.382 ± 0.00^ab^

Note: *Results are shown as the mean ± standard deviation of three iterations (n = 3) with different letters a–h in the same column (P < 0.05) (Duncan's New Multiple Range Test).

The water activity of all the samples was determined using a water activity analyzer (series 3 TE; AquaLab, CA, USA). The results showed that samples dried for 6 h had water activity from 0.374 ± 0.00 to 0.382 ± 0.00 with non-significant differences among drying temperatures of 50, 60, and 70 °C. Based on these results, the drying time required to obtain the lowest water activity in duckweed should be at least 6 h.

This was because a longer drying time led to greater water extraction from the sample. Thus, a large amount of water evaporated. Consequently, samples with longer curing times were drier and had the lowest moisture content. This process inhibits the growth of various microorganisms, including mold, yeast, and bacteria, which helps to extend the shelf life. Therefore, when selecting the conditions for drying duckweed samples, it is important to select low-temperature conditions to reduce energy consumption and maintain the nutritional value of the food.

Table 7 shows the results of the comparison of the protein and bioactive compounds obtained from the three samples: flavonoid and total chlorophyll contents of all three samples, with sample 14 as the control. The amount of protein analyzed using the Kjeldahl method was greater than that analyzed using the Biuret method. The protein content analyzed using the Kjeldahl method was between 54.579 ± 0.77 and 56.737 ± 1.04%, while the protein content analyzed using the Biuret method ranged from 30.802 ± 3.82 to 57.230 ± 3.05%. These results indicate that an increase in temperature and time causes the protein to lose its natural condition, resulting in a reduced amount of protein. The total phenolic flavonoid, and chlorophyll contents were between 51.678 ± 2.58 and 58.321 ± 3.74 μgGAE/g, 145.951 ± 5.18 and 165.357 ± 5.72 μgCatechin/g, and 22.325 ± 0.37 to 23.975 ± 0.18 mg/g dry weight, respectively. The total phenolic content of the samples in this study was higher than that previously reported [1] at 21.14 mg/g dry weight, including the total chlorophyll, which was lower than that previously reported [1] at 30.17 mg/100 g dry weight.

**Table 7:** The % of protein and bioactive substances in dried duckweed sample 12 under different temperature and drying conditions.

Condition		% Protein^KM^ (Dry basis)	% Protein^BM^ (Dry basis)	Total Phenolic (μ gGAE/g dry weight)	Total Flavonoid (μ gCatechin/g dry weight)	Total Chlorophyll (mg/g dry weight)
Temperature	Time					
**50**	4	55.711 ± 0.19^ab^	30.802 ± 3.82^a^	55.085 ± 1.32^abcd^	161.685 ± 6.63^ac^	23.838 ± 0.15^ab^
	5	55.783 ± 0.25^ab^	42.208 ± 3.46^b^	52.379 ± 2.00^ab^	147.246 ± 3.26^a^	23.335 ± 0.15^ab^
	6	55.752 ± 0.08^ab^	35.075 ± 1.22^a^	55.278 ± 1.35^bcd^	159.844 ± 6.65^bc^	22.908 ± 0.15^ab^
**60**	4	54.968 ± 0.85^a^	31.514 ± 5.36^a^	58.321 ± 3.74^d^	160.371 ± 5.24^bc^	23.975 ± 0.18^b^
	5	55.639 ± 0.23^ab^	43.781 ± 1.82^b^	51.678 ± 2.58^a^	145.951 ± 5.18^a^	22.903 ± 1.00^ab^
	6	56.737 ± 1.04^b^	34.353 ± 1.98^a^	56.527 ± 2.80^cd^	165.357 ± 5.72^c^	22.736 ± 0.39^a^
**70**	4	55.614 ± 0.95^ab^	41.914 ± 2.50^b^	53.520 ± 2.04^abc^	152.042 ± 4.49^ab^	22.325 ± 0.37^a^
	5	54.579 ± 0.77^a^	41.130 ± 3.32^b^	54.685 ± 3.53^ab^	159.999 ± 5.75^bc^	23.026 ± 0.62^ab^
	6	55.327 ± 0.20^ab^	34.079 ± 1.94^a^	54.998 ± 1.81^abcd^	162.717 ± 3.98^c^	22.682 ± 1.27^a^

Note: *Results are shown as the mean ± standard deviation of three iterations (n = 3) with different letters a–h in the same column (P < 0.05) (Duncan's New Multiple Range Test).

KM denotes the % value of protein analyzed using the Kjeldahl method.

BM denotes the % value of protein analyzed using the Biuret method.

The bioactive content was high under low-temperature drying conditions (Table 7). The 4- and 6-h drying conditions at 50 and 60 °C resulted in more bioactive compounds, particularly at 70 °C, because the amount of bioactive substances decompose or lose their natural state, thus decreasing quantity. However, when the drying times were compared, the amount of bioactive compounds increased for periods of more than 6 h. This can be explained in terms of the high temperature for a long period, causing the binding of phenols (bound phenolics) to slowly decompose from other binding molecules, resulting in higher amounts of bioactive compounds.

## 4 Conclusion

This study examined the cultivation and drying conditions of duckweed to determine the optimum conditions for preserving its nutritional value and bioactive compounds. The findings indicated that sample 12 showed the most favorable drying conditions and contained significant amounts of protein, fat, crude fiber, ash, and carbohydrates. Duckweed was grown under aquaculture conditions in an outdoor cement pond with hydroponic EC 0.5 mS/cm, which resulted in the highest protein content.

Furthermore, the results showed that a drying temperature of 50 °C and a duration of 6 h were the optimum conditions for sample 12. This resulted in high total phenolic, flavonoid, and chlorophyll contents in the dried product. HPLC analysis also revealed the presence of phenolic compounds, including protocatechuic and coumaric acids, in duckweed.

In summary, this study determined that dried duckweed plants grown under aquaculture conditions in an outdoor cement pond with hydroponic EC 0.5 mS/cm and dried under optimum conditions contained substantial amounts of protein, total phenolics, total flavonoids, and total chlorophyll. These bioactive compounds make duckweed a viable ingredient in functional health food products.

## Author contribution statement

Kakanang Posridee: Performed the experiments; Analyzed and interpreted the data; Wrote the paper.

Ratchadaporn Oonsivilai, Anant Oonsivilai: Conceived and designed the experiments; Analyzed and interpreted the data; Wrote the paper.

Arak Tira-aumphon: Contributed reagents, materials, analysis tools or data.

Jittra Singthong, Jirawan Oonmetta-aree: Analyzed and interpreted the data.

## Data availability statement

Data will be made available on request.

## Declaration of competing interest

The authors declare that they have no known competing financial interests or personal relationships that could have appeared to influence the work reported in this paper.
